# A bibliometric analysis of PIN1 and cell death

**DOI:** 10.3389/fcell.2022.1043725

**Published:** 2022-10-31

**Authors:** Jia-Heng Zhang, Shi-Yu Ni, Ya-Ting Tan, Jia Luo, Shu-Chao Wang

**Affiliations:** ^1^ Clinical Medicine Five-year Program, 19 Grade, Xiangya School of Medicine, Central South University, Changsha, China; ^2^ Center for Medical Research, The Second Xiangya Hospital of Central South University, Changsha, Hunan, China; ^3^ Department of Anatomy and Neurobiology, School of Basic Medical Sciences, Central South University, Changsha, Hunan, China; ^4^ Hunan Key Laboratory of the Research and Development of Novel Pharmaceutical Preparations, Changsha Medical University, Changsha, China

**Keywords:** bibliometric analysis, Pin1, cell death, apoptosis, autophagy, necrosis, CiteSpace, VOSviewer

## Abstract

**Background:** Regulation of cell death plays a key role in numerous diseases. As a proline isomerase, prolyl cis-trans isomerase NIMA-interacting 1 (Pin1) is important for the regulation of signaling pathways. An in-depth understanding of how Pin1 participates in the process of cell death, which affects the occurrence and development of diseases, will aid in the discovery of new disease mechanisms and therapeutic methods. Thus, the purpose of our study was to discover the research trends and hotspots of Pin1 and cell death through bibliometric analyses and to provide insights for understanding the future development of basic research and treatment of diseases.

**Methods:** Documents were extracted from the Web of Science Core Collection on 7 May 2022. We selected articles and reviews published in English from 2000 to 2021, and visual and statistical analyses of countries, institutions, authors, references and keywords were performed using VOSviewer 1.6.18 and CiteSpace 5.8.

**Results:** A total of 395 articles and reviews were selected. Since 2001, the number of articles on Pin1 and cell death has increased annually. Publications come from 43 countries, with the US having the most publications and citations. We identified 510 authors, with Giannino Del Sal having the most articles and Paola Zacchi having the most co-citations. *The Journal of Biological Chemistry* is the most researched journal, and *Nature* and its subjournals are the most cited journals. Apoptosis, phosphorylation, and breast cancer were the three most common keywords.

**Conclusion:** The number of documents showed an increasing trend from 2001 to 2014. Stagnant growth after 2014 may be related to the absence of new research hotspots. Cooperative links between core institutions need to be strengthened, and the institution with the highest citation count in recent years is Fujian Medical University in China. The role of Pin1 in cell death requires further research to discover new research hotspots. Before breakthroughs in molecular mechanism or signaling pathway research, future research will focus more on the treatment of diseases represented by Pin1 inhibitors.

## Introduction

Prolyl cis-trans isomerase NIMA-interacting 1 (Pin1) is an 18-kDa protein with an N-terminal WW domain and a C-terminal PPIase domain (Kumari et al., 2021; Tonnus et al., 2021; Zheng et al., 2021). The WW domain of Pin1 binds to phosphorylated serine-proline or threonine-proline (pSer/Thr-Pro) motifs, followed by PPIase domain isomerization of the prolyl bond in the pSer/Thr-Pro motifs (Koikawa et al., 2021; Kumari et al., 2021). In contrast, the activity of mitotic and nuclear proteins is regulated by Pin1 in a phosphorylation-dependent pathway (Kumari et al., 2021; Wang et al., 2023).

Pin1 plays a role in multiple types of cell death, such as apoptosis, necrosis, and autophagy. The effects of Pin1 on apoptosis include inactivation of Bcl-2-associated X protein (Bax), subsequently increasing the anti-cell death capacity of Bcl-2 (Bianchi et al., 2019; Makinwa et al., 2020) as well as promoting the expression of pro-apoptotic genes represented by p53 in cells to initiate apoptosis (Balaganapathy et al., 2018; Hleihel et al., 2021). This dual pro- and anti-apoptosis feature provides researchers with a variety of options for finding treatment options for diseases based on different pathways (Balaganapathy et al., 2018; Hleihel et al., 2021). Recent studies have shown that cellular necrosis (traditionally thought to be characterized by membrane disruption, release of internal substances, and inflammatory cells) also shows molecular-level abnormalities (Chen et al., 2021a; Hu et al., 2021a; Theobald et al., 2021; Tonnus et al., 2021), such as pyroptosis (Lammert et al., 2020) and neutrophil death (Sung and Hsieh, 2021). For example, Pin1 exerts a positive regulatory effect on calpain-2 by inhibiting calpastatin (CAST) in the Pin1-CAST-calpain pathway involved in cell-regulated necrosis (Cheng et al., 2018; Wang et al., 2018). This further mediates the transfer of apoptosis-inducing factors to the nucleus, ultimately leading to DNA degradation (Wang et al., 2018; Wang et al., 2019b). In a study on Pin1 and autophagy, Wang et al. (2023) found that GSK-3β is involved in autophagy as a mediator that regulates Pin1 activity.

The function of Pin1 is tightly regulated at multiple levels under physiological conditions, which makes Pin1 very different from most other constitutively active PPIases (Lu et al., 2007; Zhou and Lu, 2016). Moreover, abnormalities in Pin1 are involved in the occurrence and progression of neurological diseases and cancer through many mechanisms. Thus, further research on Pin1 can provide clues for exploring the treatment of neurological diseases and cancer. Our lab focuses on the study of Pin1 and cell death, and we hope to analyze the existing research on Pin1 in a broader field. Bibliometrics, a discipline that applies mathematical and statistical methods to the literature, provides us with a useful tool to broaden our research horizons (Smith, 2008). Using visual analysis software packages such as CiteSpace, researchers can achieve qualitative and quantitative evaluations of research progress in specific subject areas of research (Chen, 2006). Other visualization software packages such as VOSviewer, can achieve similar functions. However, the two software packages have their own characteristics. Flexible use, alone or in combination, in different situations can achieve better results. There are many evaluation indicators for bibliometric analysis, including citation frequency, the number of publications, and centrality (Avena and Barbosa, 2017).

The purpose of our study was to discover and analyze the current status and future trends of Pin1 and cell death. Using bibliometrics to identify current research hotspots in this field and explore future research trends can promote our understanding of the role of Pin1 in the process of cell death and provide references for researchers to broaden their ideas in basic research and disease treatment.

## Data and methods

### Data strategy and selection criteria

**FIGURE 1:**
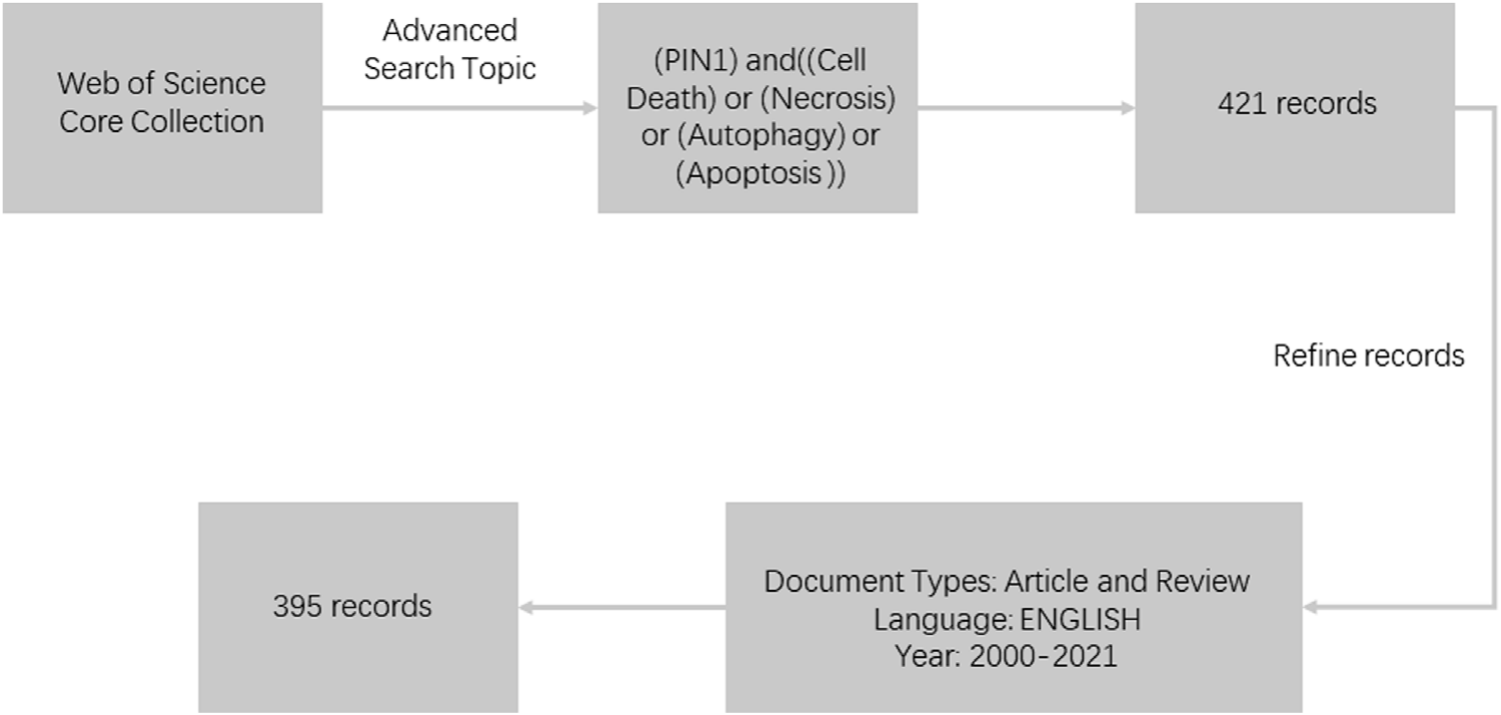
Flowchart of literature selection.

After searching the MeSH Database (https://www.NCBI.nlm.nih.gov/mesh) for relevant keywords, we searched the Web of Science Core Collection using the following search strategy: All={(PIN1) and [(Cell Death) or (Necrosis) or (Autophagy) or (Apoptosis)]}. After obtaining the search results, we found it was not until 2001 that a large number of articles related to cell death appeared. Therefore, we decided to narrow the time range to 2000–2021, and focus our study on more relevant articles. We set the language type to English, selected only Article and Review, and excluded conference materials, books, editing materials, corrections, letters, and retracted papers. Finally, 395 relevant documents were obtained for bibliometric analysis. Documents were exported as all records and references, saved as plain text files (txt) for bibliometric and visual analysis (Figure 1). The literature search was finally completed on 7 May 2022.

### Methodology

The content of bibliometric analysis includes publication year, country and region, institution, journal, author, keywords and key references. We imported all data extracted from the Web of Science Core Collection into VOSviewer (version 1.6.18; https://www.vosviewer.com/downloavosviewer) and CiteSpace (version 5.8. R3; https://sourceforge.net/project/citespace/files/latest/do) for bibliometric analysis and visualization.

CiteSpace is a citation visualization analysis software that focuses on analyzing the potential knowledge contained in scientific analysis. It has gradually developed under the background of scientometrics and data visualization. Since the structure, law and distribution of scientific knowledge are presented through visualization, the visualization graphs analyzed of such methods are called “Scientific knowledge graph”.

VOSviewer is a program for building and viewing bibliometric graphs. It can build a visual map of authors or journals, or a keyword map, based on the data. The main purpose of using VOSviewer is to analyze bibliometric networks and build visual network graphs, ultimately achieving a deep and comprehensive understanding of the structure and dynamic development of scientific research (van Eck and Waltman, 2010).

We used Microsoft Office Excel 2019 to analyze and visualize the relationship between the number of articles published and time. The parameters of VOSviewer are set as follows: Method (Linlog/Modular). The parameters of CiteSpace are set as follows: clustering method (LLR), time slice (2000–2021), number of years per slice (1), etymology (select all).

## Results

### Distribution of publications by year

**FIGURE 2:**
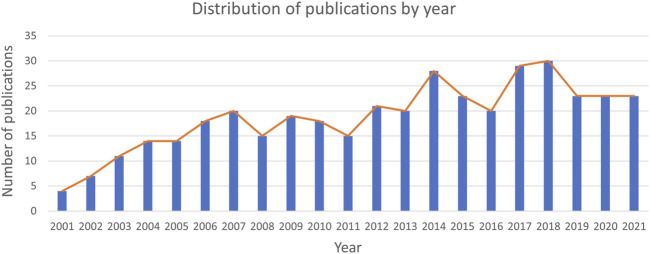
Trends of publications over the past 20 years. From 2001 to 2014, the number of documents showed an increasing trend, stabilized after 2014, and peaked in 2018.

Of the 395 publications that were analyzed, 302 were articles (76.46%) and 85 (21.52%) were reviews. The developmental trend of Pin1 research in the field of cell death can be understood from the publication time in the literature. The distribution of publication times in the literature is shown in Figure 2, where the quantity of literature shows an unstable overall growth trend over time. From 2001 (*n* = 4, 1.01%) to 2011 (*n* = 15, 3.80%), the number of studies increased but stabilized at 20 or less every year. From 2012 (*n* = 21, 5.06%) to 2021 (*n* = 23, 5.82%), the growth was erratic although the number of studies exceeded 20. Data show that while the research in this field received a certain amount of attention, it did not present a research hotspot limited to a relatively lower number of published studies. This shows that study in this field is not in high demand. Improving the emphasis in this field and making greater research efforts may show good prospects for future research.

### Countries and regions

**FIGURE 3:**
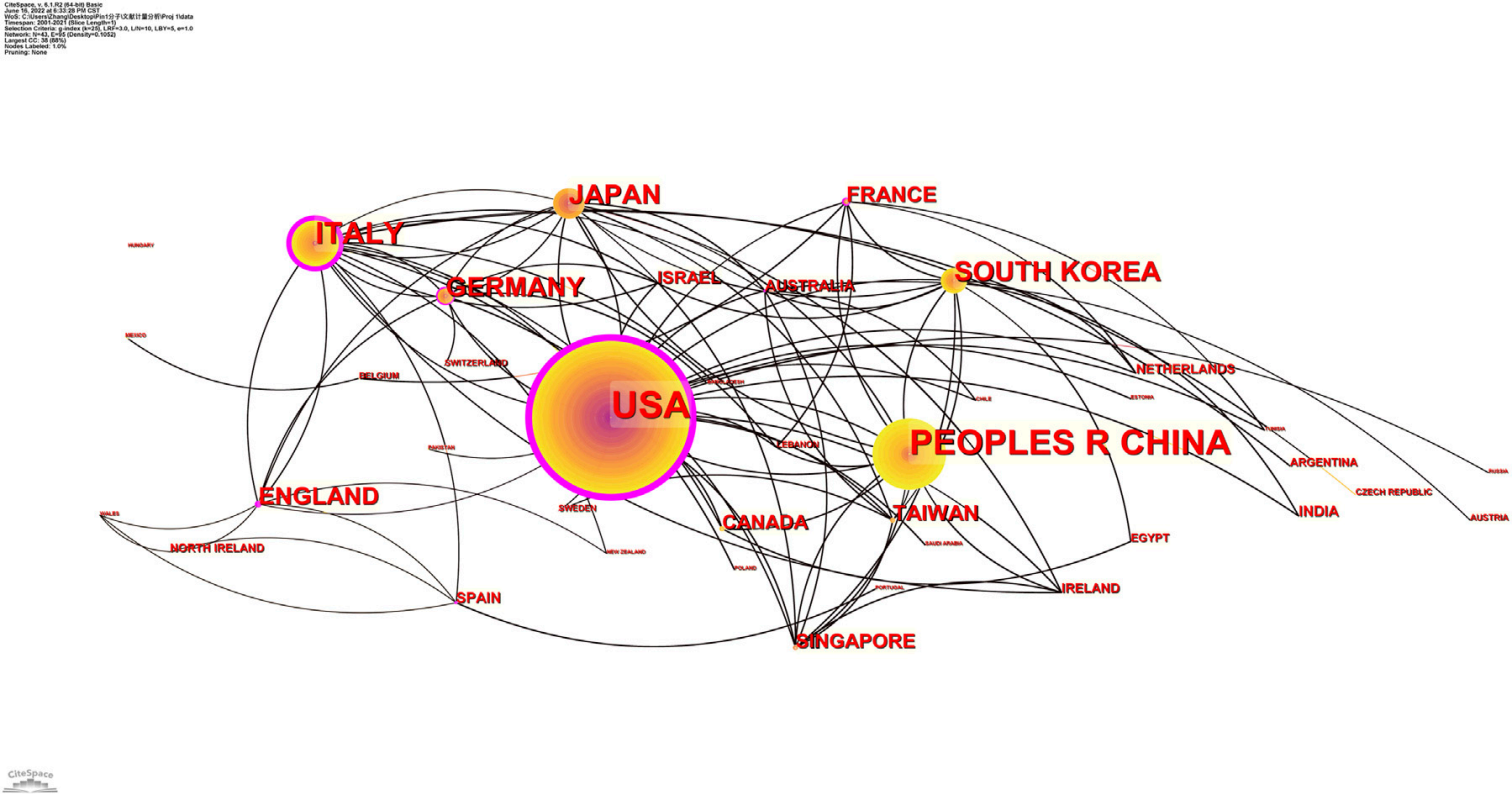
Distribution of publications from different countries/regions. The country with the largest number of publications is the United States, which also has the highest centrality of all countries. China has the second highest number of publications, but a lower centrality.

**FIGURE 4:**
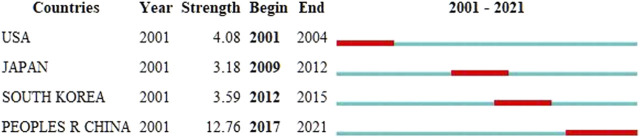
Top four countries/regions with the strongest citation bursts. A variable experiencing large changes in a short period of time indicates a strong burst of citations, and the red bar indicates the duration of the burst.

Research teams from 43 countries and regions published 395 papers. In the distribution map of publishing countries, each circle represents a country. The size of the circle represents the publication outputs of the country, and the line between the circles represents cooperation between countries (Figure 3). Centrality describes the importance of a country or region represented by this node in this research field. The top ten countries and regions with the most literature are listed in Table 1. The country with the most literature is the United States (*n* = 147, 37.22%), followed by China (*n* = 91, 23.04%) and Canada (*n* = 54, 13.67%) (Figure 3). The country with the highest centrality in the literature is the United States (0.78), followed by Italy (0.43) and England (0.20) (Figure 4).

**TABLE 1:** Top 10 most productive countries and regions with publications.

Countries/Regions	Count	Citation	Centrality	Total link strength	First published year
United States	147	14,963	0.78	91	2001
China	91	2,459	0.03	37	2006
Italy	54	2,897	0.43	38	2002
South Korea	34	829	0.09	23	2005
Japan	33	2,955	0.05	26	2002
Germany	30	1,662	0.17	21	2001
England	21	993	0.20	16	2001
France	15	688	0.17	10	2002
Taiwan	14	710	0.01	13	2002
Canada	11	710	0.04	5	2006

### Organizations

**FIGURE 5:**
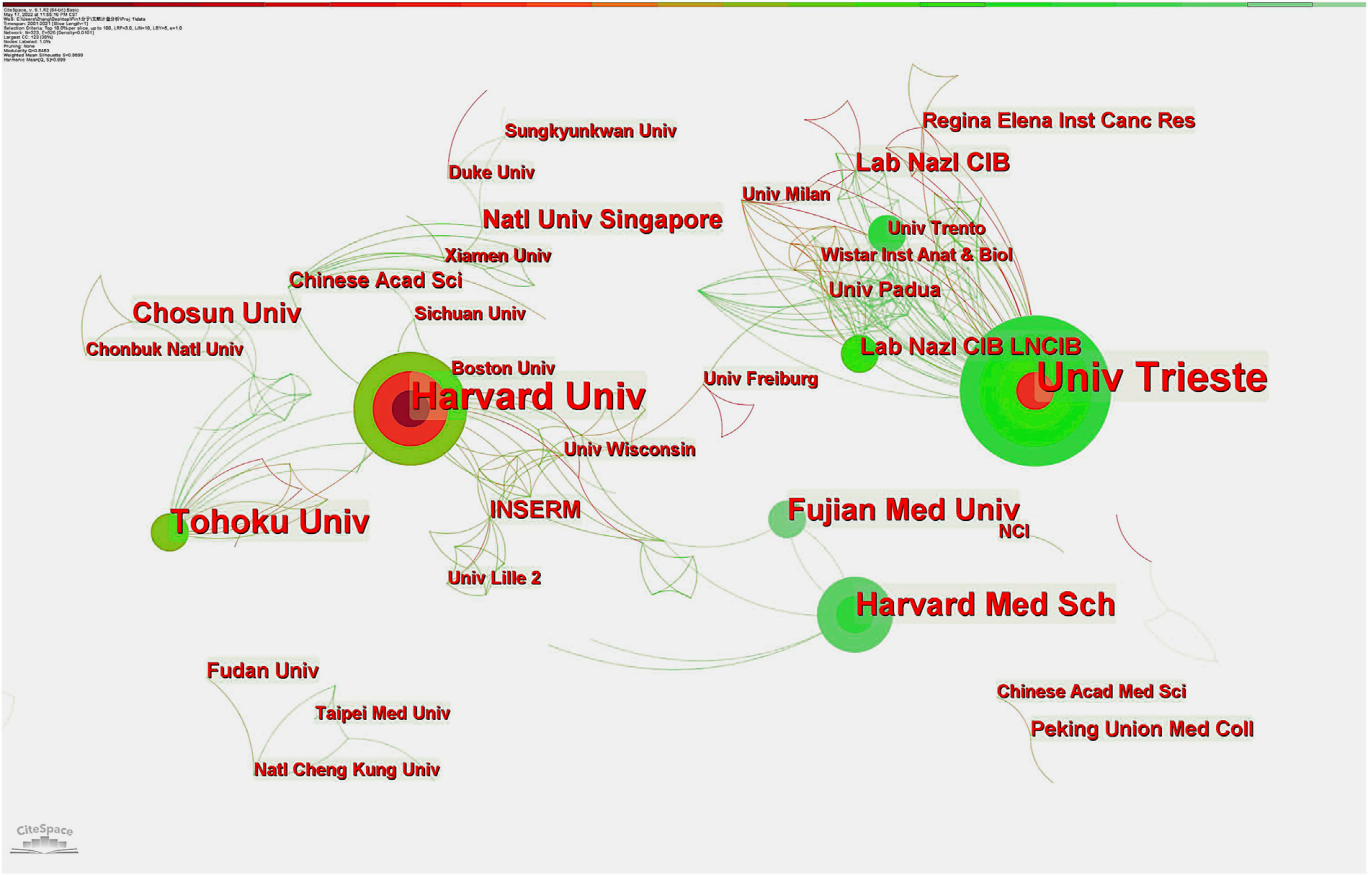
Distribution of publications from different institutions. Harvard University and the University of Trieste are tied for first place in the number of publications, with Harvard having the strongest centrality. These two core agencies have more cooperative links with most other institutions.

According to an analysis of CiteSpace and VOSviewer, 395 studies were published by 397 institutions. The top ten institutions with the most prolific publications are listed in Table 2. The most prolific institutions were University of Trieste (*n* = 22, 5.57%) and Harvard University (*n* = 22, 5.57%), followed by Tohoku University (*n* = 14, 3.54%). Of the top 10, two were American and two Italian. The most cited institutions include Harvard University (3,563), University of Trieste (2,234), Tohoku University (1,648), and Lab Nazl CIB (944); the co-occurrence relationship is shown in Figure 5. The evidence above indicates that institutions in the United States and Italy are leading research on Pin1 in the field of cell death.

**TABLE 2:** Top 10 most productive organizations.

Rank	Organization	Documents	First published year	Centrality	Citations	Country/Regions
1	University of Trieste	22	2002	0.16	2,234	Italy
2	Harvard Univ	22	2001	0.24	3,563	United States
3	Tohoku Univ	14	2002	0.15	1,648	Japan
4	Harvard Med Sch	11	2016	0.04	274	United States
5	Fujian Med Univ	9	2017	0.01	177	China
6	Chosun Univ	9	2010	0.00	167	South Korea
7	Lab Nazl CIB	8	2002	0.04	944	Italy
8	INSERM	8	2002	0.04	355	France
9	Natl Univ Singapore	7	2007	0.03	243	Singapore
10	Natl Cheng Kung Univ	6	2003	0.07	359	Taiwan

### Journals

**FIGURE 6:**
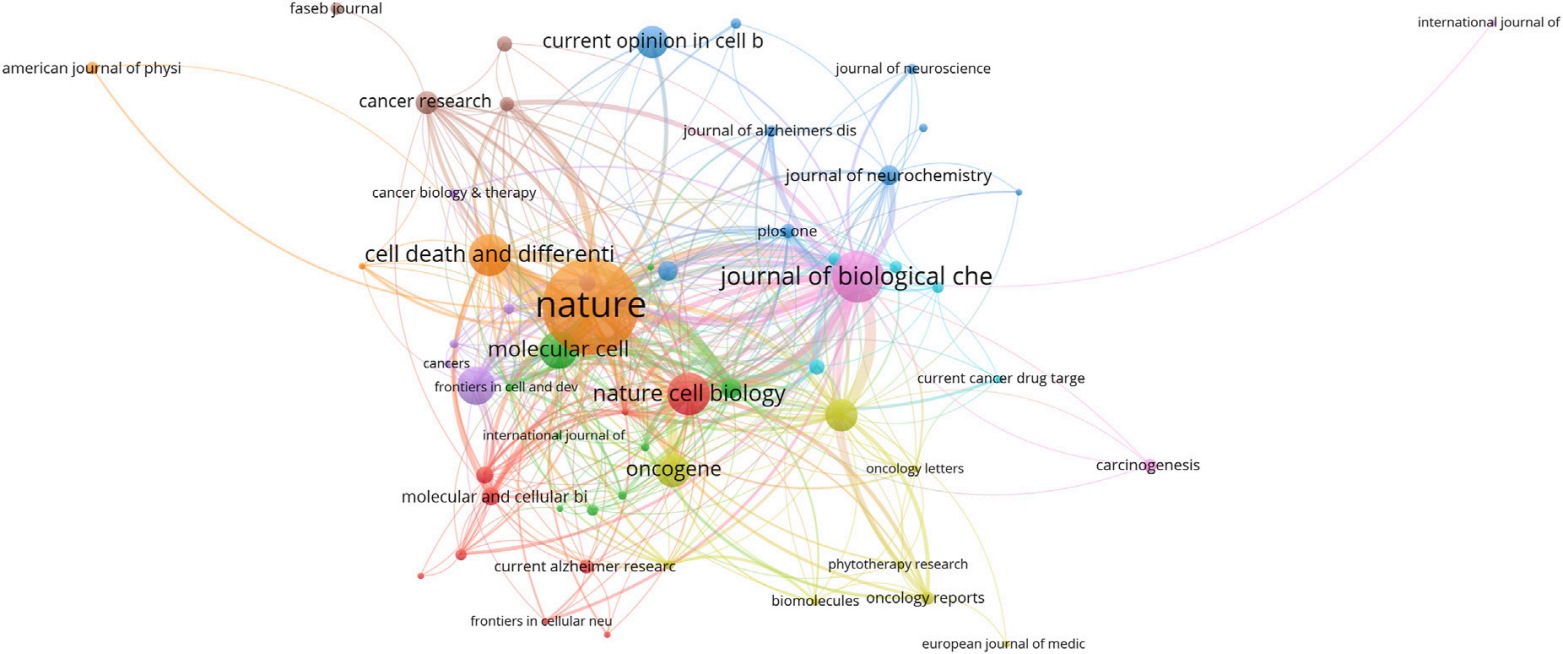
Journals with occurrence relations shown as an overlay graph plotted with VOSviewer 1.6.14. The analysis method is Linlog/modularity. The Weights was citations. The width of the line indicates the strength of the relationship.

Based on the data visualization analysis using VOSviewer, the literature on Pin1 in the field of cell death from 2000 to 2021 was mainly distributed in 221 different journals, among which 64 published more than two articles. As shown in Table 3 and Figure 6, the journals with the highest citations are *Nature* and *Journal of Biological Chemistry*, which published 4 and 21 papers, respectively, and received 5,012 and 1,569 citations, respectively. This shows that the impact factors of the top 10 journals in 2021 range from 49.962 to 5.157. In 2021, *Nature* had the highest impact factor, while *the Journal of Biological Chemistry* had the lowest. Nature is the most influential journal in this research field based on the number of citations and journal impact factor data.

**TABLE 3:** Top 10 most cited journals.

Rank	Journals	Citation	Documents	Total link strength	IF (2021)	JCR
1	Nature	5,012	4	165	49.962	Q1
2	Journal Of Biological Chemistry	1,569	21	176	5.157	Q2
3	Nature Cell Biology	1,041	4	81	28.824	Q1
4	Cell Death and Differentiation	1,010	12	92	15.828	Q1
5	Molecular Cell	866	8	71	17.970	Q1
6	Proceedings Of the National Academy of Sciences of The United States of America	857	6	42	11.205	Q1
7	Oncogene	711	8	48	9.867	Q1
8	Current Opinion in Cell Biology	637	2	13	8.382	Q1
9	Embo Journal	611	2	73	11.598	Q1
10	Cancer Research	326	7	36	12.701	Q1

### Authors

**FIGURE 7:**
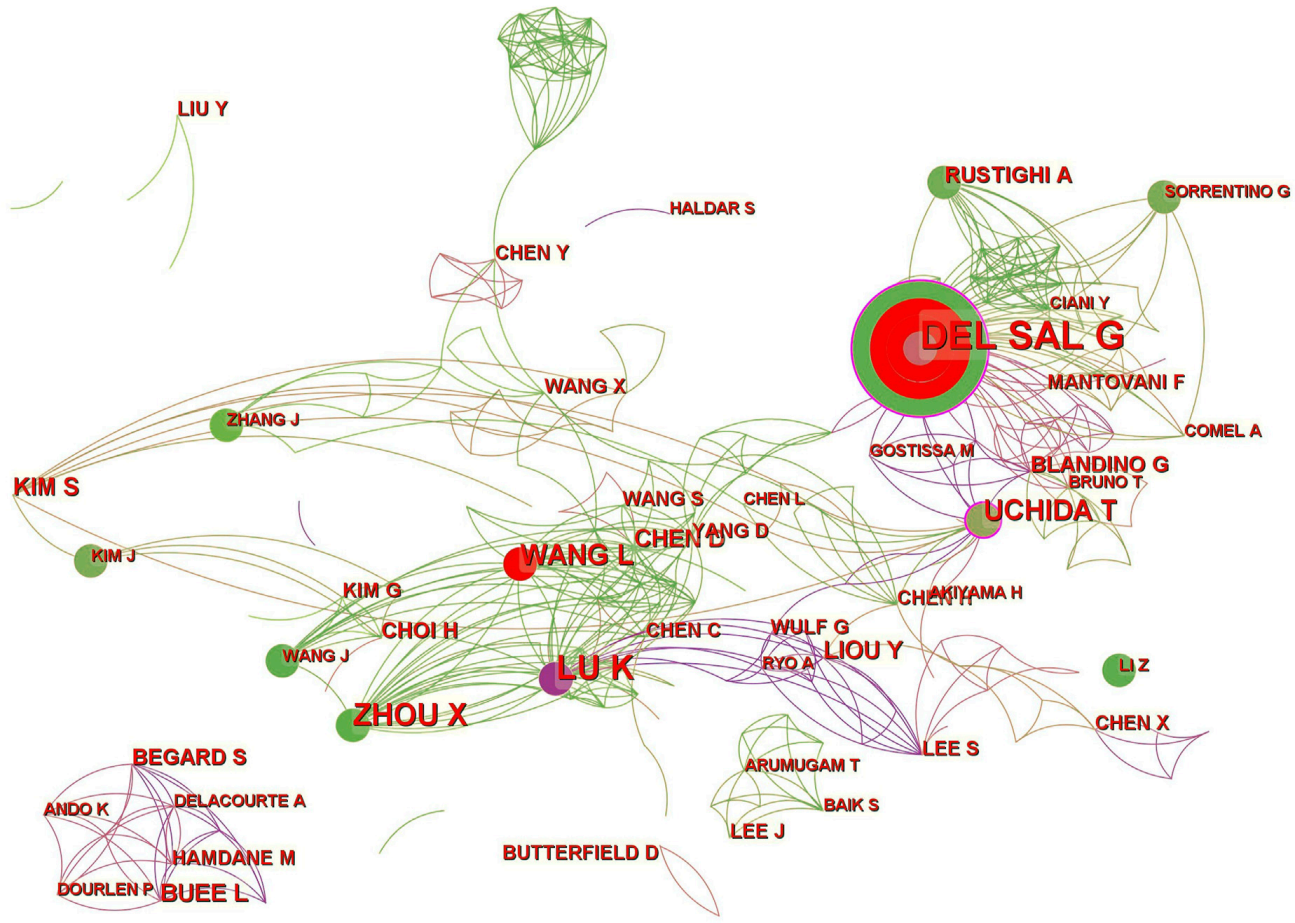
CiteSpace visualization map of authors involved. A circle represents an author, and the size of the circle represents the author’s number of articles. The purple part of the circle indicates the author’s centrality in the field.

A total of 510 authors participated in the writing of 395, studies. Collaborative clusters among different authors are shown by contact networks of distinct colors. The evaluation criteria for primary authors included the number of publications and H-index. The top 10 authors who published the most literature on Pin1 in the field of cell death from 2000 to 2021 are listed in Table 4. Giannino Del Sal of Lab Nazl CIB (LNCIB) ranks first among all authors, while Kun Ping Lu of Fujian Medical University ranks second with 15 publications. This shows the pattern of prolific authors from core institutions. The primary authors were from Italy, China, Japan, and Singapore. Figure 7 shows the patter of collaboration between authors. From the visualization diagram, we can observe that apart from a small number of author groups, author collaboration is centered on influential authors; the relationship between author groups is remarkably close. In terms of centrality, Giannino Del Sal tops the list with 0.12, and his citation number is also the highest among all the authors (*n* = 1,221), which shows that Giannino Del Sal has an important influence in this field of research; so does Kun Ping Lu.

**TABLE 4:** Top 10 most productive authors.

Rank	Authors	Documents	H-index	Centrality	Organization	Citation
1	Giannino Del Sal	26	58	0.12	Lab Nazl CIB LNCIB	1,221
2	Kun Ping Lu	15	63	0.05	Fujian Medical University	686
3	Xiaozhen Zhou	11	47	0.01	Fujian Medical University	725
4	Long Wang	9	9	0.01	Fujian Medical University	144
5	Takafumi Uchida	9	31	0.10	Tohoku University	265
6	Danna Chen	6	18	0.01	South China University of Technology	63
7	Yih-Cherng Liou	6	31	0.03	National University of Singapore	63
8	Luc BUEE	6	67	0.00	University de Lille	61
9	Seong-Jin Kim	6	40	0.04	Seoul National University	56
10	Severine Begard	5	23	0.00	University de Lille	56

### Keywords

**FIGURE 8:**
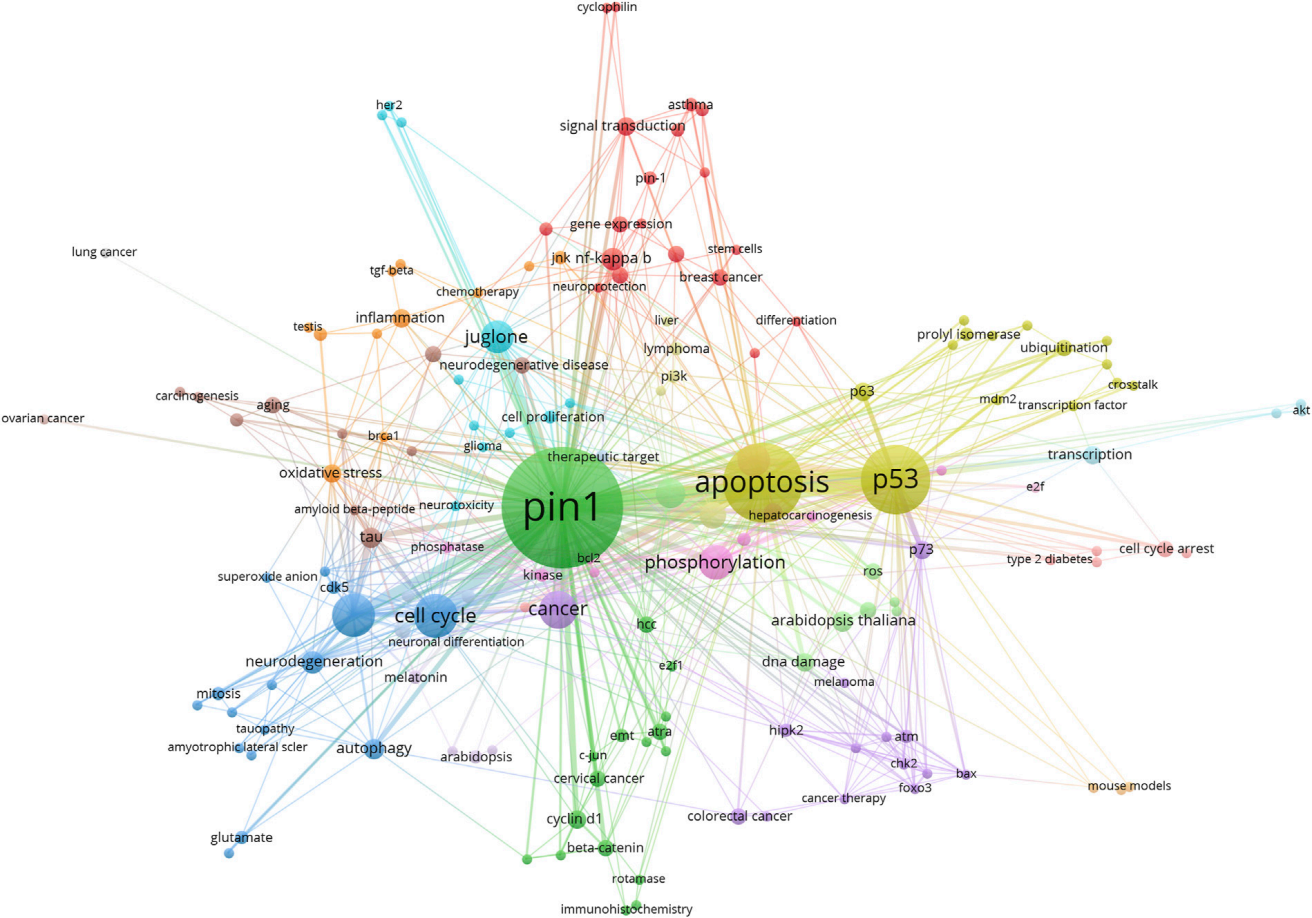
VOSviwer visualization map of keywords clustering analysis. The analysis method is Linlog/modularity. The size of the circle is the number of times the keyword appears. The colors of the circles in the figure represent the categories the keywords are in.

Keywords can prompt or express the characteristics of the topic content of this study. By analyzing the frequency and distribution of keywords, we can understand the hot trends in research. A total of 903 keywords were retrieved from 395 articles. VOSviewer was used to draw the knowledge graph of the keywords (Figure 8). The frequencies and centralities of the keywords are listed in Table 5. As shown in Table 5, high-frequency keywords were Pin1 (206), apoptosis (80), phosphorylation (78), breast cancer (63), cell cycle (48), Alzheimer’s disease (37), and p53 (30). Figure 8 shows the frequency of keywords through the size of the circle and the clustering of keywords through the circle color. Owing to the difference in statistical methods between the two software tools, the keyword rankings in Figure 8 and Table 5 are somewhat different. It also should be noted that the “prolyl isomerase Pin1” and “Pin1” in Figure 8 and Table 5 are the same, which is due to the limitation of literature measurement tools.

**TABLE 5:** Top 10 most productive keywords.

Rank	Keywords	Occurrence	Centrality	Rank	Keywords	Occurrence	Centrality
1	prolyl isomerase pin1	206	0.19	11	cell death	37	0.17
2	apoptosis	80	0.16	12	cancer	33	0.06
3	phosphorylation	78	0.13	13	p53	30	0.05
4	breast cancer	63	0.15	14	pin1	27	0.05
5	activation	56	0.13	15	gene expression	24	0.12
6	expression	55	0.10	16	mechanism	23	0.07
7	cell cycle	48	0.19	17	binding	21	0.03
8	protein	43	0.15	18	nf kappa b	21	0.09
9	dna damage	39	0.14	19	umor suppressor	20	0.03
10	alzheimers disease	37	0.13	20	*in vivo*	20	0.04

### Co-cited references and reference burst

**FIGURE 9:**
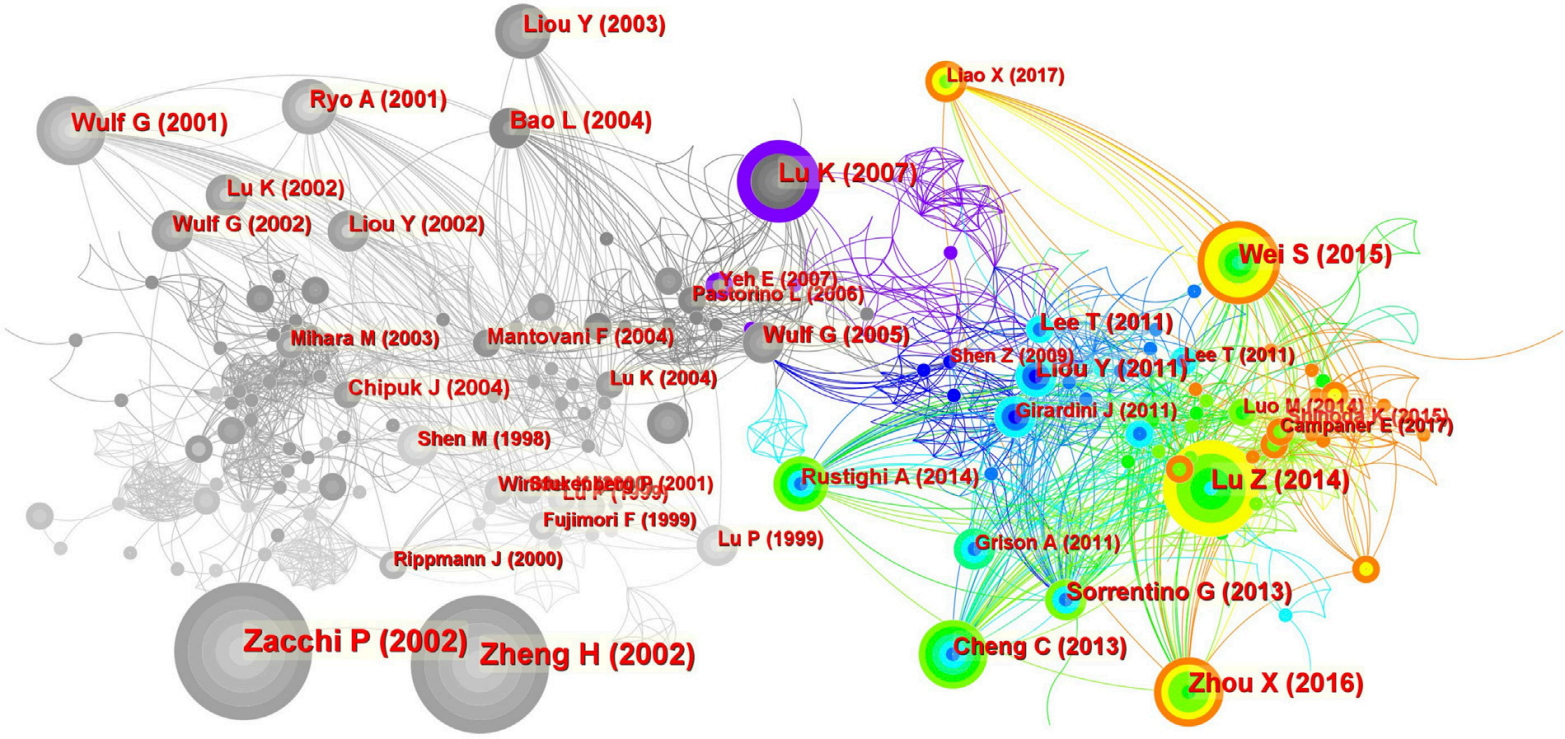
Co-citation analysis of references. The size of the circle represents the number of co-citations. The color of the circle represents the publication time of the co-cited literature, and the color of the outer circle represents the centrality of the literature.

Co-citation refers to the fact that two (or more) papers are simultaneously cited by one or more subsequent papers, and the two papers are said to constitute co-citation relationships. The number of citations reflects the field’s influence. A total of 827 co-cited references were analyzed by co-clustering, and the top 10 highly cited references are listed in Table 6. As shown in Figure 9, the authors of the most frequently cited studies are Zacchi, Zheng, Zhou, Wei, and Lu.

**TABLE 6:** Top 10 highly co-cited documents.

Rank	Title	First author	Journals	Publication year	Citations
1	The prolyl isomerase Pin1 reveals a mechanism to control p53 functions after genotoxic insults	Paola Zacchi	Nature	2002	53
2	The prolyl isomerase Pin1 is a regulator of p53 in genotoxic response	HW. Zheng	Nature	2002	50
3	The isomerase PIN1 controls numerous cancer-driving pathways and is a unique drug target	Xiao Zhen Zhou	Nature Reviews Cancer	2016	30
4	Active Pin1 is a key target of all-trans retinoic acid in acute promyelocytic leukemia and breast cancer	Shuo Wei	Nature Medicine	2015	29
5	Prolyl isomerase Pin1 in cancer	Zhimin Lu	Cell Research	2014	28
6	Development of Three-Dimensional Hollow The prolyl isomerase PIN 1: a pivotal new twist in phosphorylation signaling and disease	Kun Ping Lu	Nature Reviews Molecular Cell Biology	2007	26
7	The prolyl-isomerase Pin1 activates the mitochondrial death program of p53	G. Sorrentino	Cell Death And Differentiation	2013	23
8	Prolyl isomerase Pin1 as a molecular switch to determine the fate of phosphoproteins	Yih-Cherng Liou	Trends In Biochemical Sciences	2011	22
9	Pin1 is overexpressed in breast cancer and cooperates with Ras signaling in increasing the transcriptional activity of c-Jun towards cyclin D1	GM. Wulf	Embo Journal	2001	22
10	PIN1 Inhibits Apoptosis in Hepatocellular Carcinoma through Modulation of the Antiapoptotic Function of Surviving	Chi-Wai Cheng	American Journal of Pathology	2013	19

**FIGURE 10:**
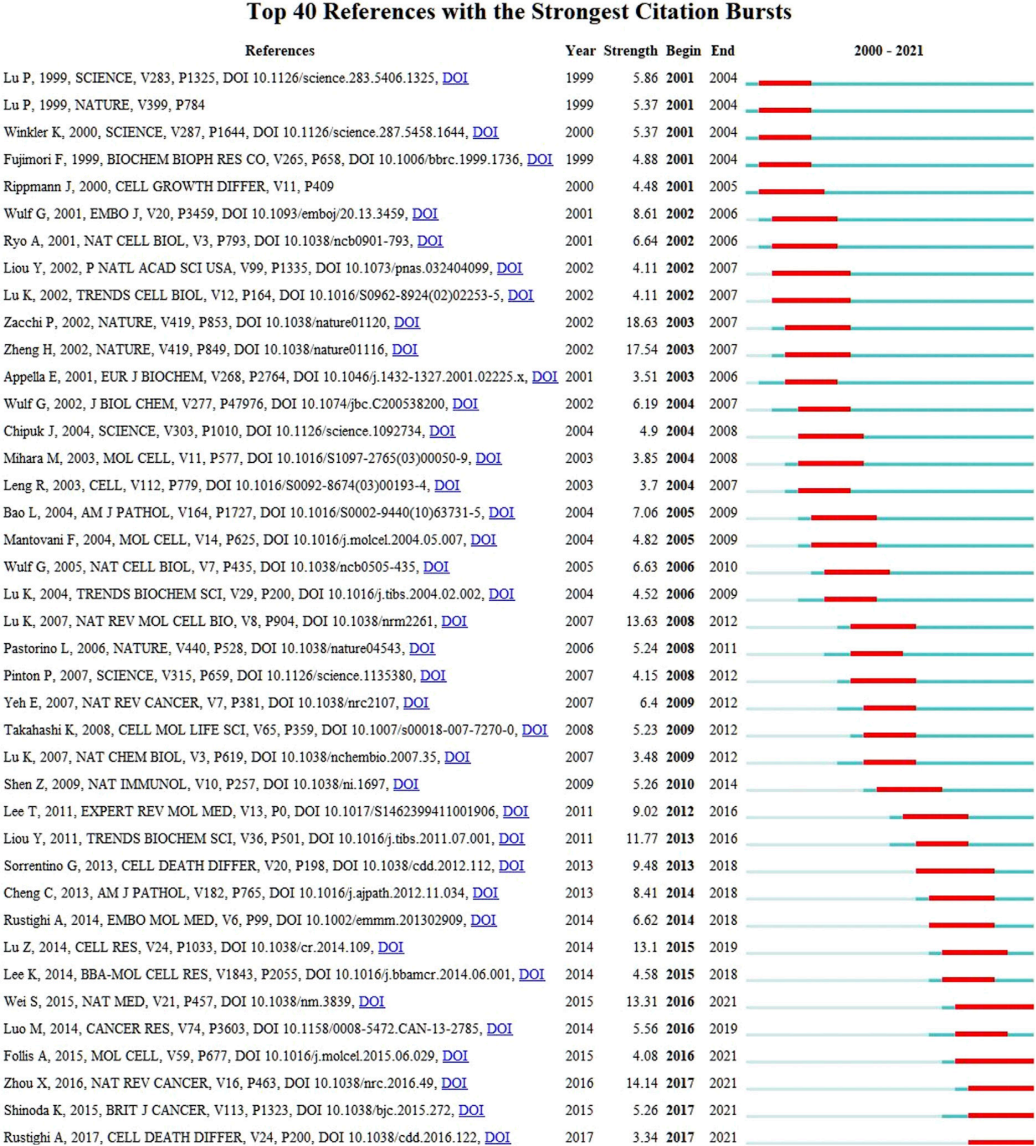
CiteSpace visualization map of top 40 references with the strongest citation bursts. A variable experiencing large changes in a short period of time indicates a strong burst of citations, and the red bar indicates the duration of the burst.

A citation explosion refers to the fact that the number of citations of a paper increases rapidly in a brief period, indicating that the paper has received considerable attention from researchers in this field during this period. Figure 10 shows 40 articles with citation outbreaks. One of the two articles with high outbreak intensity cited in the last 2 years is Shuo Wei’s “Active Pin1 is a Key Target of All-trans Retinoic acid in acute Promyelocytic leukemia and Breast Cancer” in 2015. The outbreak intensity was 13.31, and the citation outbreak lasted from 2016 until present. Another study is “The Isomerase PIN1 Controls Numerous Cancer-Driving Pathways and is a Unique Drug Target,” published by Zhou and Xiao Zhen in 2016 with an outbreak intensity of 14.14. The citation outbreak lasted from 2017 to the present. At the same time, these two papers were also the fourth and third most cited papers, respectively, indicating that these two papers have had the greatest influence on Pin1 in the research field of cell death in recent years. After analyzing Figure 10, we found that almost all hot articles with high citation outbreaks after 2013 were related to cancer; representative cancers included breast and liver cancer. The high-citation burst articles before 2013 were more related to the molecular signaling mechanism of Pin1. This further verifies our thinking about the development trends in this field.

## Discussion

### General information

The 395 studies published in the past 20 years generally showed a trend of gradual increase over time and reached a peak in 2018 (Figure 2). From 2001 to 2014, research on Pin1 was in the stage of emerging increase, and the number of studies showed a growing trend. However, since 2014, research in this field has lost the trend of growth, which indicates that this field has not received much attention, or breakthrough research progress has not been made; thus, new research hotspots have not been formed. In conclusion, previous studies have improved the upstream and downstream molecular mechanisms and signaling pathways of Pin1 in cell death, and more studies on how to reduce or delay the occurrence of diseases by inhibiting Pin1 or blocking signaling pathways are needed.

According to the distribution of countries and regions in Table 1, the number of studies published (147) and cited (14,963) in the United States is the highest and far greater than that of other countries. Centrality is used to measure the importance of the nodes in the network. The relatively important countries with a threshold of over 0.1 are the United States (0.78), Italy (0.43), and England (0.20). In addition, the United States was the first country to publish literature in this field in 2001. All the above data show that the United States is the most influential country in this field. It is worth noting that China has the second-largest number of studies. Although centrality is low, the intensity of the literature explosion in China from 2017 to 2021 is as high as 12.76 (Figure 4). It is predicted that China will play a more significant role in this field in the future.

Harvard University in the United States has the highest number of literature publications and centrality. As can be seen from the co-occurrence figure (Figure 5), the cooperation within the cluster of regional research institutions is intricately linked and centrality is obvious. This means that Harvard University, the University of Trieste, and other research centers are taken as research centers, but there are few cooperative relationships between the clusters of research institutions. For example, there is little cooperation between Harvard University and the University of Trieste, which indicates that there is great space for development in cooperation between different countries, regions, and institutions in this field. According to the outbreak trend chart of research institutions, the outbreak intensity of Fujian Medical University in China has been as high as 4.17 since 2017, which indicates that Fujian Medical University has made many achievements in this field in recent years.

Giannino Del Sal (26, 6.6%) was the author of most publications, followed by Kun Ping Lu (15, 4.1%) and Xiaozhen Zhou (11, 2.78%). P Zacchi ranks first among the most co-cited authors, and HW Zheng ranks second. Both authors’ highly co-cited articles reveal the relationship between Pin1 and P53, and they are the founding authors of research in this field. The third most frequently cited article was published by Xiao Zhen Zhou and Kun Ping Lu in Nature Reviews on Cancer. In particular, Professor Kun Ping Lu found that over-activation of Pin1 in cancer interferes with the balance of oncogenes and tumor suppressors, thereby promoting the development of cancer and cancer stem cells. In addition, the role of Pin1 in cancer and the potential of Pin1 inhibitors to restore this balance have been discussed (Zhou and Lu, 2016). In 2018, Kozono et al. found that arsenic oxide inhibits Pin1 carcinogenesis by inhibiting and degrading Pin1. Additionally, they found that all-trans retinoic acid inhibits leukemia, breast cancer, and liver cancer by targeting isomerase Pin1 (Kozono et al., 2018). Among the top 10 co-cited articles, there are many studies on Pin1 and cancer. This indicates that research on Pin1 in the field of cell death has gradually shifted to the treatment of diseases represented by Pin1 inhibitors.

According to Table 3 and Figure 6, the most frequently cited journals in the field of Pin1 and cell death were Nature (4, 1.0%) and its subsidiary Nature Cell Biology (4, 1.0%), with a total of 6,053 citations. The Journal of Biological Chemistry (21, 5.3%) published the most articles with 1,569 citations, followed by Cell Death and Differentiation (12, 3.0%) with 1,010 citations. Of the top 10 journals, nine were classified in the Q1 region. According to the results, studies on Pin1 in cell death were mostly distributed in highlyinfluential journals, indicating that research achievements in this field have received significant attention.

### Hotspots and frontiers

Based on the analysis of keyword frequencies, popular research trends can be acknowledged. As shown in Table 6, in addition to PIN1, the most common keywords were apoptosis (80), phosphorylation (78), breast cancer (63), cell cycle (48), Alzheimer’s disease (37), and p53 (30). The keywords mainly focused on cell death, molecular mechanisms, and diseases. Pin1 is involved in three aspects of cell death: apoptosis, regulatory necrosis, and autophagy.

Apoptosis is controlled by several apoptotic genes and characterized by regulated cell death (Bai et al., 2020; Hu et al., 2021b; Nascimento et al., 2022). Pin1 has been reported to mediate neuronal apoptosis by promoting the expression of pro-apoptotic genes such as p53 (Grison et al., 2011; Balaganapathy et al., 2018). In Huntington’s disease (a neurodegenerative disease), the mutated Htt protein promotes the activation of Pin1, which subsequently interacts with p53 and triggers the dissociation of its inhibitors (inhibitors of p53’s apoptotic stimulator protein; IASPP). This ultimately promotes the expression of a series of apoptotic genes and leads to cell death (Grison et al., 2011). Recent studies have shown that DAPK1, which plays a key role in cell death, is a key modulator of Pin1 activity. The reduction in Pin1 activity is accompanied by dapK1-mediated increased expression of CIS PT231-Tau, which promotes neuronal cell death (Kim et al., 2021; Qiu et al., 2021). Knockout or functional inhibition of Pin1 almost completely prevented caspase-3 activation, DNA disruption, and cell death induced by 1-methyl-4-phenylpyridinium. This suggests that Pin1 promotes apoptosis (Ghosh et al., 2013). Other studies have shown that Pin1 binds to and stabilizes the NICD1 domain through its WW domain (Baik et al., 2015) and enhances its stability by inhibiting the F-box and WD repeat domains (containing polyubiquitination mediated by domain protein 7). This ultimately results in NICD1-induced apoptosis (Wang et al., 2014; Baik et al., 2015). The pro-apoptotic effect of Pin1 is also reflected in the activation of NICD1, which is involved in the regulation of p53 transactivation and the upregulation of pro-apoptotic genes (such as Bax) (Balaganapathy et al., 2018). Reactive oxygen species (ROS) production is a key effector of the PIN1-notch-p53 pathway (Li et al., 2019). The JNK pathway plays a complex role in many physiological and pathological processes such as apoptosis and differentiation and may be activated by various stress signals and inflammation (Zhang et al., 2021). The JNK pathway also promotes mitochondrial apoptosis by regulating the release of Bax under the control of Pin1 (Shen et al., 2009). As research on mitochondria, cell necrosis, and apoptosis is becoming increasingly important (Yan et al., 2021), it is necessary to determine the role of Pin1.

Regulatory necrosis (RN) includes necrotizing apoptosis induced by tumor necrosis factor, which is mediated by receptor-interacting protein three; scoria, which is dependent on caspase-1 activation and triggered by microbial infection; aerastin-mediated iron death, which requires excess iron; and RN, which is dependent on mitochondrial permeability transition (Yuan et al., 2019; Alu et al., 2020; Chen et al., 2021b; Liao et al., 2021; Tonnus et al., 2021). Pin1 acts as an important downregulatory factor in dapK1-induced excitatory toxic necrosis (Wang et al., 2018). In addition, activation of calpain-2 induces the cleavage of apoptosis-inducing factors, which are transferred to the nucleus and induce DNA degradation, resulting in neuronal RN (Wang et al., 2018; Wang et al., 2019b). Calpastatin (CAST) is an endogenous specific inhibitor of CAST (Wang et al., 2014; Cheng et al., 2018), and Pin1 promotes excitatory toxic glutamate injury. This results in a reduced inhibitory effect of CAST on calpain-2 and induction of cell necrosis (Wang et al., 2018; Wang et al., 2019a). In recent years, PANoptosis, which has been shown to exist in neuronal ischemia/reperfusion injury (Yan et al., 2022; Yan et al., 2023), and other new regulatory modes of necrosis (such as iron necrosis and copper necrosis) have been discovered successively. However, Pin1 has not received much attention in these fields, and the relevant regulatory mechanisms still need to be further studied.

Autophagy plays a critical role in cell homeostasis by degrading dysfunctional and degraded proteins and damaged organelles inside cells into autophagosomes, which fuse with lysosomes to form autolysosomes (Chao et al., 2022). Pin1 is thought to regulate the proteasome during autophagy (So and Sum, 2015). Pin1 inhibits GSK-3β expression by inhibiting proteasome degradation, and ultimately stimulates cell death through autophagy (So, 2015). In contrast, inhibition of GSK-3β induces Pin1 activation, suggesting that GSK-3β may be a mediator of Pin1 activity in autophagy (Chao et al., 2022).

## 5 Limitations

Our literature retrieval work was completed on 7 May 2022, and the retrieval date ranged from 1 January 2000, to 31 December 2021. Therefore, it is possible that some articles that met the retrieval conditions could not be retrieved because their publication dates were too early or too late, making the number of studies in our database different from the actual number. Only articles and reviews were selected for this study because we wanted to control the quality of the included literature as much as possible. However, through this process, we might have ignored the research progress from other publications that have higher values. The fields of Pin1 and cell death cover many aspects of research, among which many primary keywords were not included in the retrieval formula. This made it impossible to retrieve more relevant research results, despite helping us avoid importing irrelevant articles. In addition, because of the limitation of tools, we can only search the literature on the Web of Science Core Collection; thus, the literature not included in the Web of Science Core Collection is omitted. Finally, bibliometric tools, such as CiteSpace and VOSviewer, still have limitations in their functions. They cannot replace systematic scientific retrieval and are still limited by the experience and knowledge level of researchers.

## 6 Conclusion

Research on the molecular mechanism between Pin1 and cell death, the difference between Pin1 functions in different signaling pathways, and the role of Pin1 in the prevention or delay of diseases is of great research value, especially has exciting application prospects in the field of cancer and nervous system diseases. We used bibliometrics to analyze and visualize the current situation of research in this field and found that research on Pin1 and cell death shows an increasing trend over time, and that there are opportunities for further research. Undoubtedly, the United States plays the most vital role in this field, and Harvard University is the most influential institution. China’s outstanding performance in recent years is sufficient to keep us hopeful that China will play a more key role in this field in the future. Professor Kun Ping Lu is an outstanding researcher in this field of study in that he and his team have published several valuable research results in high-influence journals. Another sign that research on Pin1 and cell death has attracted more attention is that many research results in this field have been included and cited by internationally influential journals. We speculate that pending the discovery of new molecular mechanisms or signaling pathways, future studies on Pin1 and cell death will focus more on the treatment of diseases.

## Data Availability

The original contributions presented in the study are included in the article/supplementary material, further inquiries can be directed to the corresponding author.
